# Interspecific Aggression and Habitat Partitioning in Garter Snakes

**DOI:** 10.1371/journal.pone.0086208

**Published:** 2014-01-22

**Authors:** Michael Edgehouse, Leigh C. Latta, Edmund D. Brodie, Edmund D. Brodie

**Affiliations:** 1 Department of Natural Science and Mathematics, Lewis-Clark State College, Lewiston, Idaho, United States of America; 2 Department of Biology, Reed College, Portland, Oregon, United States of America; 3 Department of Biology and Mountain Lake Biological Station, University of Virginia, Charlottesville, Virginia, United States of America; 4 Department of Biology, Utah State University, Logan, Utah, United States of America; University of Plymouth, United Kingdom

## Abstract

Defense of a limited resource, such as space or food, has recently been discovered in snakes and has been widely documented in lizards. Garter snakes (*Thamnophis spp.*) are historically considered generalist predators such that food is not a limiting resource. However, in this study we show that the common garter snake (*Thamnophis sirtalis*) and the aquatic garter snake (*Thamnophis atratus*) show a strong preference for amphibians as their primary food source at the Santa Lucia Preserve (SLP), Monterey County, California. This food preference forces these snake species at SLP to exploit aquatic habitats. Our principle goal was to investigate the aggressive behavior of *T. sirtalis* and the potential that this aggression displaces *T. atratus* from its preferred habitat. We found that when individuals from either species are alone, a 100% preference for aquatic or near aquatic habitat is observed. In contrast, when these species are together, *T. sirtalis* occupy the aquatic habitat and *T. atratus* occupy an area far removed from water. *Thamnophis sirtalis* often physically force *T. atratus* from the aquatic habitat through repeated biting and other displays of aggression.

## Introduction

Aggressive interactions within or between species that arise from competition for a limited resource are an important ecological mechanism structuring communities [1]. Numerous communities of vertebrates, such as desert rodents, salamanders, and lizards, are structured by both intra- and interspecific aggressive interactions [2]–[9]. Conspicuously absent from those vertebrates in which aggression is an important determinant of community structure are snakes. There is evidence that intraspecific aggression, such as competition for a limited resource [8] and male-to-male combat in response to limited female availability [9]–[12], can shape the population structure of some snake species. However, in snakes, food partitioning typically shapes the community structure of sympatric snake populations [13]–[17], while there is no evidence that interspecific aggression is found among communities of snakes.

Snake communities can segregate or coexist on the basis of intra- or intergeneric interactions. A community of *Thamnophis sirtalis* and *Coluber constrictor* coexisted based on food partitioning [13] (*T. sirtalis* preying mainly on birds and *C. constrictor* preying mainly on rodents). Information on the diet of a species often provides information on the preferred habitat of an organism when direct observation is not possible [18]–[19]. Additionally, diet data are informative for other aspects of an organism such as growth, resource utilization, and potential competition between closely related species [20]. Another mechanism that shapes snake communities is pre-emptive competition. For example, sympatric *Thamnophis spp.* may have increased competition based on similarities in body size, diet, and general habits [15]. Often this pre-emptive competition for space or food can lead to segregation or local extinction [4], [5], [13], [16], [17].

This study focuses on snake communities at the Santa Lucia Preserve (SLP), Monterey Co. CA. Santa Lucia Preserve is a locality where *T. sirtalis*, *T. atratus* and *T. elegans* coexist with an abundant, toxic prey item, the California newt (*Taricha torosa)*. *Thamnophis sirtalis* and *T. atratus* from SLP are aquatic and demonstrate independently evolved elevated levels of resistance to tetrodotoxin (TTX) [21], a potent neurotoxin, found in the skin of newts that binds to voltage gated sodium channels (reviewed by [22]–[24]). Additionally, during collections at SLP it was noted that *T. sirtalis* were found near water at certain locales while *T. atratus* were found far removed from water at these same locales. In contrast, *T. atratus* were found at the water's edge only at ponds without *T. sirtalis*, suggesting that the habitat utilization of *T. atratus* depends on the presence of *T. sirtalis*.

The similarities in the physiology (resistance to TTX) of these species suggests that they utilize similar prey items, however the drastic change in habitat utilization by *T. atratus* in the presence of *T. sirtalis* suggests that *T. sirtalis* is actively displacing *T. atratus*.

## Methods

### Animal Collection

Snakes were collected by hand at the following locations throughout the SLP; 36°26.24.32 N 121°47.33.43 W, 36°26.001 N 121°44.664 W, 36°27.741 N 121°46.876 W, 36°27.179 N 121°46.684 W, 36°26.21.85 N 121°46.49.68 W, 36°27.27.19 N 121°47.58.07 W. All of the collection localities had the same principle conditions of a water source surrounded by vegetation. Upon collection, animals were forced to regurgitate by stomach palpation [25], measured for snout-vent length (SVL), weighed and uniquely scale clipped for identification. Animals were then housed individually in bags in a cooler at 20 C and watered once a day for five days prior to the start of each behavioral trial. Housing snakes individually for five days insured that all animals used in the trials were exposed to the same conditions prior to the start of each behavioral trial. We do not believe that the collection of natural history data nor the ventral clipping affected the behavior trials.

### Experimental Methods

**Figure 1:**
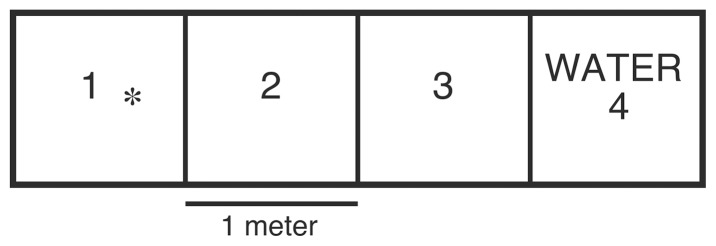
Behavior test chamber diagram. Enclosures were 4- meter delineations, by 1 meter wide, by .45 meters high. Each 1-meter zone was assigned numbers 1, 2, 3, or 4. Zone 1 was farthest from water and contained one 30 cm×30 cm×1 cm Styrofoam cover object (indicated by an asterisk), zone 4 was in the water. Snakes were introduced into the enclosures in zone 1.

Ten 4 m×1 m×0.45 m enclosures made with black, half centimeter weatherproof nylon mesh were utilized to determine microhabitat preference. Enclosures were marked each meter and set 1 m in water and 3 m out of water. One cover object (0.3 m×0.3 m×12.5 mm Styrofoam®) was provided on the ground in the meter farthest from the water (zone 1; Fig. 1). Each trial consisted of a 24-hour period with one animal in the enclosure. Snakes were placed in the enclosure at 0700 hours, six observations of position, movement, and behavior were recorded for each snake during each trial. Data were recorded at 0800, 1000, 1200, 1400, 1600 and 1800 hours, from a distance of approximately 20 m to avoid observer influence; observations were repeated as above for trials with two snakes in the chamber. All observations during trials were made using binoculars by the same individual (ME). At each observation, the snake's position was recorded as 1 (zone farthest from water) 2, 3, or 4 (zone in water; Fig. 1), depending upon the zone in which the majority of the snake's body was located. If an animal was on the border between two zones, the head of the snake was used to determine the zone. Instances of aggressive behavior directed toward the heterospecific (during trials with more than one snake), such as biting, chasing, head flattening, and hissing were recorded. Observations of initial hissing were removed from analysis because of possible observer influence, subsequent observations of hissing were made by observing behavior similar to that noted during initial hissing observations; this behavior has been classified as head flattening for analysis. Biting often occurred as 2–3 directed strikes occurring rapidly in a row. Each sequence of strikes was counted as 1 bite. More than one aggressive behavior was recorded as observed. For example, an obvious head flattening followed by 3 strikes was counted as 1 head flatten and 1 bite. Trials were conducted on the southeast corner of a managed wetland at SLP (36°26.25.39 N, 121°47.35.19 W). This site was chosen to conduct behavior trials because shallow water enabled the observer to position himself at multiple angles to the enclosure 20 meters away.

Animals were assigned to trials matching SVL as close as possible (Table 1). Twenty *T. sirtalis* vs. *T. atratus* trials were conducted starting on 06 May 2005. These trials consisted of placing one *T. sirtalis* in each enclosure for 24 hours and recording observations as described. Upon completion of this 24-hour period a *T. atratus* was added and six observations were again recorded for 11 hours as described above. Because we could not collect enough snakes, and because we did not use any individual in more than one trial, 13 *T. atratus* vs. *T. sirtalis* trials matching sex (all male) and SVL as closely as possible were conducted starting on 26 May 2006. These trials consisted of placing one *T. atratus* in each enclosure for 24 hours and recording observations as described. Upon completion of this 24-hour period a *T. sirtalis* was added and observations were again recorded for 11 hours. Subjects were used in only one set of trials. Two sets of 10 control trials with either *T. sirtalis* or *T. atratus* used as both focal and introduced species were conducted following the same design.

**Table 1:** Descriptive statistics for snakes used in behavior trials.

	Trial			
	SVL		Mass	
Species	TSTA	TATS	TSTA	TATS
TS	70.5 (4.9)	60.0 (4.8)	172.8	93.7
TA	52.8 (4.6)	51.5 (2.6)	81.1	79.0
df	24	24	24	24
t	5.29	3.09	5.15	.862
p	*<0.001*	*0.006*	*<0.001*	.*397*

Means (±2 SE) of SVL and mass for snakes in the heterospecific trials (TSTA trial where *T. sirtalis* was the focal species and *T. atratus* the species added; TATS trial where *T. atratus* was the focal species and *T. sirtalis* the added species) with associated statistics for two sample t-tests assuming unequal variances. In both trials *T. sirtalis* displayed significantly higher SVL than *T. atratus*. TS indicates *T. sirtalis*; TA indicates *T. atratus*.

### Diet

Snakes were collected by hand throughout SLP between 05 May 2004 and 27 September 2006. Upon capture, each animal was SVL measured, weighed, given a unique ventral scale clip for future identification, and forced to regurgitate by stomach palpation [25]; each food item was identified. Subjects were then released at the site of capture.

### Ethics Statement

The study was observational and subjects were held a minimum length of time for the purpose of this study; each individual was used only once. No individual was injured. Research was approved by the Utah State University Institutional Animal Care and Use Committee, Protocol #1008. We thank we thank California Department of Fish and Game for providing scientific collecting permits (801071–05).

## Analysis

### Habitat Preference

To assess how the presence of a conspecific or heterospecific snake influences habitat preference in the focal snake species we analyzed data at each of six sampling intervals for three groups that describe the position of the focal subject, conspecific, or heterospecific snake. The groups were: 1) the position of the focal snake alone in the enclosure, a measure of the habitat preference of the focal species; 2) the position of the focal snake in the enclosure in the presence of the conspecific or heterospecific species, a measure of the habitat preference of the focal species in the presence of a conspecific or heterospecific; and 3) the position of the conspecific or heterospecific subject in the enclosure in the presence of the focal, a measure of the habitat preference of the conspecific or heterospecific.

In order to account for the non-normality and heteroscedasticity in our data, and the fact that we have repeated measures on the same subjects, we used the Friedman test to assess statistical differences between the three groups we defined previously [26]. The Friedman test is a non-parametric test for differences among levels of a grouping factor when there is also a blocking factor with multiple levels that may contribute to variation in the levels of a group. For our analysis, the grouping factor is snake species/combination (three levels) and the blocking factor is the sampling interval (six levels).

If the position of the focal species alone does not differ from the position of the focal species in the presence of the conspecific or heterospecific then the conspecific or heterospecific does not alter the habitat preference of the focal species. Alternatively, if the position of the focal species alone does differ from the position of the focal species in the presence of a conspecific or heterospecific then the conspecific or heterospecific does alter the habitat preference of the focal species. All habitat preference analyses were conducted in Program R using the agricolae package [27].

One concern is that the patterns we observe may be driven by differences in body size between the focal and added snakes. In order to test whether size is an important determinant of movement between positions in the experimental chambers we first calculated two metrics that describe differences in body size and position. The first metric, size differential, was calculated by subtracting the SVL of the added species from the SVL of the focal species. The second metric, movement differential, was calculated for each time-step by subtracting the position of the focal species in the experimental chamber after the addition of the second species from the position of the focal species before the addition of the second species. We calculated these metrics for the dataset in which *T. sirtalis* was the focal species and *T. atratus* was the added species, and also for the dataset in which *T. atratus* was the focal species and *T. sirtalis* was the added species. For each dataset, we then performed regression analyses using size differential as the independent variable and movement differential as the dependent variable.

### Diet Preference

To examine the differences in diet among the snake species at SLP we used a generalized linear model assuming a Poisson distribution for the prey counts with species treated as the main effect. We obtained p-values by using a Chi-squared test to compare the reduction in deviance of the main effect to the residuals. Each of the two prey types recovered from the snake species (i.e., anurans and salamanders) were analyzed separately.

## Results

### Habitat preference

**Figure 2:**
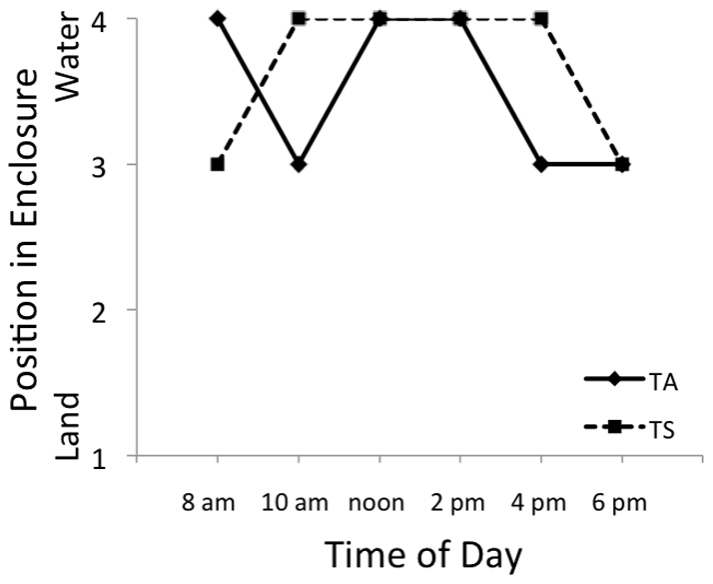
Median positions of *Thamnophis atratus* (TA) and *Thamnophis sirtalis* (TS) when alone in the enclosures at SLP. Abbreviations are the same as in figures 2 and 3.

**Figure 3:**
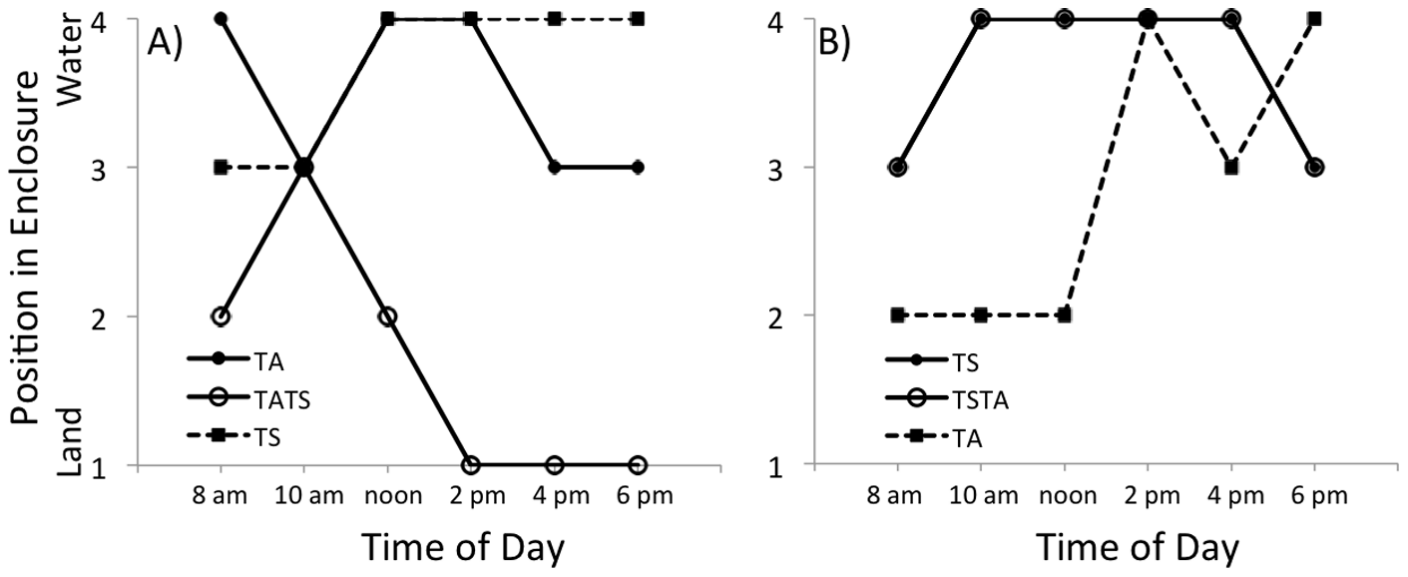
Median positions of *Thamnophis atratus* (TA) and *Thamnophis sirtalis* (TS) in enclosures for the SLP locality. The focal species before (filled circles and solid lines) and after (open circles and solid lines) the addition of the second species (squares and dashed lines) are indicated in each panel. A) The response of *T. atratus* to the addition of *T. sirtalis*. B) The response of *T. sirtalis* to the addition of *T. atratus*. Only *T. atratus* showed a significant change in position in response to the addition of *T. sirtalis*.

Both T. *sirtalis* and *T. atratus* occupied the aquatic portion of the test chamber when in the chamber alone (Fig. 2). There was a significant difference in the spatial occupation of *T. atratus* (focal species) and *T. sirtalis* (χ^2^ = 10.33; p = 0.006) when in the test chamber together. There was also a significant difference in spatial occupation of *T. atratus* when solo in the test chamber and the position of *T. atratus* when the chamber was co-occupied by *T. sirtalis* (χ^2^ = 6.00; p = 0.014). In every series of trials (13 total) with a single *T. atratus* in the test chamber, *T. atratus* preferred positions close to, or in the water (positions 3 and 4; Fig. 2 and 3). However, in the presence of *T. sirtalis,* the position of *T. atratus* shifted significantly to terrestrial habitats (positions 1 and 2; Fig. 3A). Most of the *T. sirtalis* used in these trials were observed to exhibit aggression (10 of 13). Fifteen aggressive displays (11 head flatten, 4 strikes) were observed from 10 different *T. sirtalis*; it is likely we failed to observe other aggressive interactions from the distance of 20 m. Fourteen of 15 aggressive displays were observed between 800 and 1200 hours. We saw no aggression from *T. atratus* in any trial. Additional trials alternating focal species (*T. sirtalis* solo, *T. atratus* introduced) were conducted and the results indicate no significant differences in spatial occupation by *T. sirtalis* were present in any trial (χ^2^ = 2.33; p = 0.311); when *T. sirtalis* were in the test chamber first they occupied the water, or near the water and *T. atratus* did not approach the water (or the *T. sirtalis*). There were no observations of aggression from *T. sirtalis,* as the focal snake. In the presence of conspecific snakes, neither *T. sirtalis* (χ^2^ = 1.09; p = 0.580) nor *T. atratus* (χ^2^ = 1.45; p = 0.483) altered their position in the enclosure.

In both sets of trials *T. sirtalis* was larger than *T. atratus* (Table 1). Our analyses for the effect of size on movement suggested that differences in SVL do not explain differences in movement in the experimental chamber. Neither the regression when *T. sirtalis* was the focal species, nor when *T. atratus* was the focal species, yielded significant results (*T. sirtalis*: df = 102, F = 1.614, p = 0.2069; *T. atratus*: df = 115, F = 0.28, p = 0.598).

### Diet

**Figure 4:**
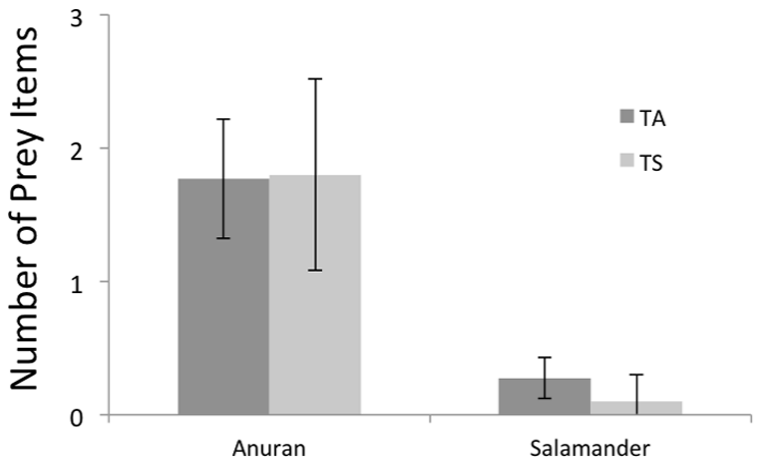
Mean number of prey (±2 SE) for *Thamnophis atratus* (TA) and *Thamnophis sirtalis* (TS) consumed by individuals at SLP.

Diets of *T. atratus* and *T. sirtalis* were similar based on generalized linear modeling (anuran: deviance = −0.005, p = 0.94; salamander: deviance = −1.34, p = 0.25; Figure 4). Stomach contents reveal that both *T. sirtalis* and *T. atratus* are amphibian specialists at this locality; 100% of the prey recovered from stomachs of both species were amphibians. The most abundant amphibian prey recovered in the stomachs and observed in the field were *Taricha torosa* and *Pseudacris regilla* (Northern Pacific Treefrog). We have no food records from *T. atratus* found away from water and cannot confirm they were feeding when in the terrestrial habitat.

## Discussion

*Thamnophis sirtalis* potentially displace *T. atratus* with aggressive behavior under experimental conditions and field observations did not detect these two aquatic species syntopically at any of the ponds at SLP. This is the first time interspecific aggression has been implicated in microhabitat distribution within snake communities. Similar to observations of kukrisnakes that defend a food resource from conspecifics by aggression [8], this defense of territory by *T. sirtalis* was often accompanied by direct physical interaction. Ten of 13 *T. sirtalis* were observed to display aggression. Fifteen aggressive displays (11 head flatten, four strikes) were observed from these 10 different *T. sirtalis.* However, in contrast to kukrisnakes, which bit conspecifics, this behavior involved physical displays of body and head flattening and typically repeated strikes towards the anterior portion of the heterospecific individual. The majority of aggressive displays were observed between 800 and 1200 hours, possibly indicating that dominance had been established.

The shift in the preferred spatial occupation of *T. atratus* from aquatic to terrestrial habitats upon addition of *T. sirtalis* indicates that a unique interaction is occurring at SLP. Both species clearly prefer aquatic habitats when alone, however, the addition of *T. sirtalis* to a chamber occupied by *T. atratus* resulted in an abrupt shift in zonal occupation by *T. atratus* from aquatic habitats to terrestrial habitat. When the experiment was conducted in reverse fashion (*T. sirtalis* focal species) no habitat displacement of *T. sirtalis* was seen. During trials in which *T. sirtalis* was the focal species, *T. atratus* did not enter into the zone occupied by *T. sirtalis* for the majority of the trial time frame. Only after *T. sirtalis* moved from zone 4 to zone 3 did *T. atratus* occupy zone 4 (Fig. 3B). These results suggest that *T. sirtalis* is the behaviorally dominant species. This conclusion is corroborated by the fact that aggressive displays were made by *T. sirtalis* toward *T. atratus*, but never by *T. atratus* toward *T. sirtalis*. We acknowledge that using a single observer limited the time frame in which observations were conducted, possibly resulting in an underreporting of aggression. In all trials, *T. sirtalis* were larger (SVL and mass) than *T. atratus.* The difference in size may have influenced the behavior of the snakes.

The similarity in diet of *T. sirtalis* and *T*. *atratus* may help explain the unique interaction we have observed. Stomach contents reveal that both *T. sirtalis* and *T. atratus* are amphibian specialists in this area, 100% of the prey recovered from stomachs were amphibians. Interspecific aggression leading to displacement is more likely to occur between biologically similar species [7]. If both, *T. sirtalis* and *T. atratus* are vying for a limited resource(s) (such as habitat and/or food); it is more likely that interspecific territoriality will occur [17]. The spatial partitioning we have seen at SLP between *T. atratus* and *T. sirtalis* may be a direct result of food availability and aggressive defense of this food source. The most abundant amphibian prey recovered in the stomachs and observed in the field were *T. torosa* and *P. regilla*. Feldman et al. 2010 showed that 100% of the *T. sirtalis* and roughly 75% of the *T. atratus* from SLP are resistant enough to not be affected by the toxin that the newts possess, making this prey a viable food source for all *T. sirtalis* and the majority of *T. atratus*. The result of this defense of a limited resource has manifested itself as the spatial occupation shift that we observe.

Summer months at SLP bring many ecological changes, including drying of grasslands and greatly reduced water levels. The changes that occur throughout the summer reduce the suitable habitat and resources for *Thamnophis sp*. If two species are ecologically similar enough and persist in a homogeneous environment with limited but definable resources, they should exhibit interspecific territoriality [28]–[29]. This hypothesis is applicable to the interactions observed at SLP. During the summer, resources (water and food) utilized by *T. atratus* and *T. sirtalis* are declining at a rapid rate, forcing the animals to occupy a more compact space; resulting in a behavior that has not been seen at other locales. Further trials at SLP are warranted to test this hypothesis and observe the long-term consequences of these behaviors. Our data confirm that *T. sirtalis* from SLP are using aggression to defend a limited resource, the toxic prey, *T. torosa*, resulting in an ecological niche shift of the behaviorally inferior *T. atratus*.

## Conclusions

The composition of *Thamnophis* populations throughout Monterey Co. CA suggest aggressive behavior is structuring snake communities there and possibly throughout the region. Levels of tetrodotoxin resistance of snakes vary throughout this region, however, the majority of snakes in Monterey Co, both *T. sirtalis* and *T. atratus*, have the ability to consume *T. torosa* as a viable food source. The factors that maintain segregated snake populations in Monterey Co. are not entirely defined, but diet and behavior data from SLP suggest that these life history aspects help maintain segregation.
